# Precise Ensemble Face Representation Given Incomplete Visual Input

**DOI:** 10.1177/2041669518819014

**Published:** 2019-01-07

**Authors:** Jason M. Haberman, Lauren Ulrich

**Affiliations:** The Department of Psychology, Rhodes College, Memphis, TN, USA; College of Health Sciences, William Carey University, Hattiesburg, MS, USA

**Keywords:** ensemble perception, faces, amodal completion

## Abstract

Humans can recognize faces in the presence of environmental noise. Here, we explore whether ensemble perception of faces is similarly robust. Is summary statistical information available from crowds of faces that are visually incomplete? Observers viewed sets of faces varying in identity or expression and adjusted a test face to match the perceived average. In one condition, faces amodally completed behind horizontal bars. In another condition, identical facial information was presented, but in the foreground (i.e., face parts appeared on fragmented strips in front of a background). Baseline performance was determined by performance on sets of fully visible faces. The results revealed that the ensemble representation of amodally completing sets was significantly better than the fragmented sets and marginally worse than in the fully visible condition. These results suggest that some ensemble information is available given limited visual input and supports a growing body of work suggesting that ensembles may be represented in the absence of complete visual information.

At a given moment, our retinas are inundated with millions of bits of information—more information than the brain can consciously represent at any one time. A host of studies on change blindness and capacity limitations of attention verify this, pointing to an extremely sparse conscious visual experience (Alvarez & Cavanagh, 2004; Luck & Vogel, 1997; Rensink, O’Regan, & Clark, 1997; Simons & Chabris, 1999; Treisman, 1982). Despite these limitations in conscious visual awareness, the scene beyond the focus of attention does not altogether vanish; *something* is available, and that something may aide in the deployment of limited attentional resources (Wolfe, 1994; Wolfe & Horowitz, 2004). It has been proposed that, in the face of overwhelming information, the visual system can exploit the redundancy of natural scenes by representing their summary statistics (Alvarez, 2011; Haberman & Whitney, 2012; Whitney, Haberman, & Sweeny, 2014), a phenomenon known as ensemble perception (Ariely, 2001). Ensemble perception is a robust and ubiquitous heuristic, operating efficiently (Alvarez & Oliva, 2009; Chong & Treisman, 2003; Haberman & Whitney, 2011) across all levels of the visual system (Haberman, Brady, & Alvarez, 2015). It is independent of single object recognition (Chong, Joo, Emmanouil, & Treisman, 2008; Whitney et al., 2014), functional under diminished attentional resources (Alvarez & Oliva, 2008), and sensitive to an array of summary statistics beyond the central moment (Dakin, 1999; Haberman, Lee, & Whitney, 2015; Solomon, 2010). Ensembles may be derived across a variety of visual domains, from oriented gabors (Attarha & Moore, 2015; Dakin, Bex, Cass, & Watt, 2009; Dakin & Watt, 1997; Parkes, Lund, Angelucci, Solomon, & Morgan, 2001) to faces varying in expression and identity (Fockert & Wolfenstein, 2009; Haberman & Whitney, 2007, 2009; Leib et al., 2014), and many of these representations appear to be mechanistically independent (Haberman et al., 2015).

Demonstrations of ensemble representations often reflect a warped memory trace, whereby observers recall summary information not ever displayed rather than individual object information actually presented (e.g., Ariely, 2001; Haberman & Whitney, 2009; Maule, Witzel, & Franklin, 2014). This may be regarded as the visual system’s attempt to efficiently and accurately summarize a complex visual scene. For example, when asked whether a test item appeared in the previously displayed set, observers will false alarm to the average item (e.g., average expression) while showing relative little awareness of the individual items composing that set (Ariely, 2001; Haberman & Whitney, 2009). Thus, it appears the visual system automatically derives information that is not explicitly displayed, a testament to the power of the ensemble heuristic. This flexibility raises an important question: How much physical information is needed to generate an accurate summary representation?

This question gets at the notion of an *amodal* ensemble—summary information in the absence of physical input. If observers can generate a precise summary representation in the presence of limited information (due to occlusion or other interference), it would support the oft-made claim that ensembles provide visual stability in a noisy and dynamic environment (Cohen, Dennett, & Kanwisher, 2016; Corbett & Melcher, 2014; Whitney et al., 2014). That is, despite impoverished visual information, ensemble perception might continue to provide veridical and valuable information about natural scenes.

There already exists some evidence that ensemble information remains available despite low-fidelity input, suggesting mechanisms are in place to overcome environmental interference (e.g., occlusion). For example, even when foveal information is obscured from view, observers can still derive precise ensemble information based on noisy peripheral input (Wolfe, Kosovicheva, Leib, Wood, & Whitney, 2015). In addition, observers can discriminate average expression information when they are unable to localize individual changes driving the average differences (Haberman & Whitney, 2011) and even when they are unable to discriminate individual expressions due to crowding (Fischer & Whitney, 2011). All this points to the possibility that ensembles may be represented amodally, that is, summary information may be available even in the absence of physical information.

In the current set of experiments, we explored amodal ensemble representation by leveraging what is already known about amodal completion, a strong cue to occlusion and scene depth (e.g., Coren, 1972; Kanizsa, 1976). In amodal completion, an object appears to complete behind an occluding surface—imagine viewing a face that is behind a set of blinds (Figure 1). Various Gestalt cues drive this percept (e.g., good continuation and good form; Wertheimer, 1923), countering the possible and valid percept of multiple discrete and discontinuous objects. With amodal completion, object identification proceeds unimpaired, despite the reduction of available information; the expression of the face depicted on the left of Figure 1 is no less identifiable than the complete, identical face on the right. Thus, amodal completion offers an elegant approach to exploring whether precise ensemble information, like individual object perception, may be derived under impoverished viewing conditions.

**Figure 1.**
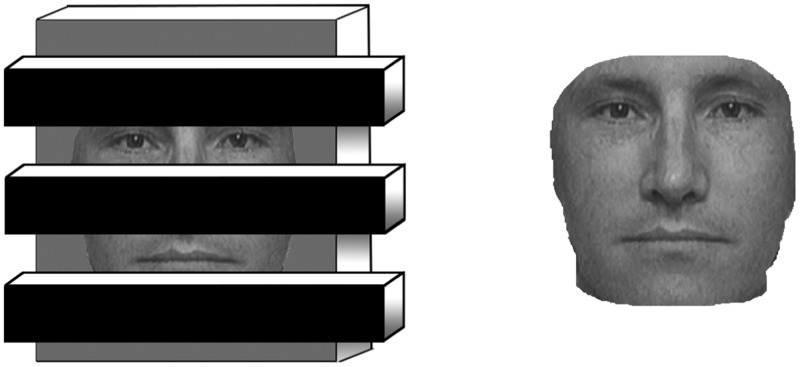
Both faces are equally identifiable, even though the left image is partially occluded by a series of blinds.

Ensemble perception is a hierarchical operation, whereby different visual domains are represented by independently operating mechanisms (Haberman et al., 2015)—in this study, we chose to focus on the domain of face perception. Ensemble face perception is surprising given the computational sophistication required for facial processing. While several studies have revealed robust support for ensemble face perception (e.g., Fischer & Whitney, 2011; Fockert & Wolfenstein, 2009; Haberman & Whitney, 2007; Leib et al., 2012, 2014; Sweeny, Grabowecky, Paller, & Suzuki, 2009), it may nonetheless rely upon having access to complete and uninterrupted visual information. If the ability to represent the average expression or the average identity of a set of faces remains unimpaired given impoverished information, however, it would suggest a critical role for the ensemble heuristic in perceptual stability. While advanced artificial visual systems still struggle to properly identify faces in the presence of noise or occlusion (Scheirer, Anthony, Nakayama, & Cox, 2014), the human visual system might overcome these perceptual limitations by *combining* noisy information across a complex scene.

## Experiment 1

**Figure 2.**
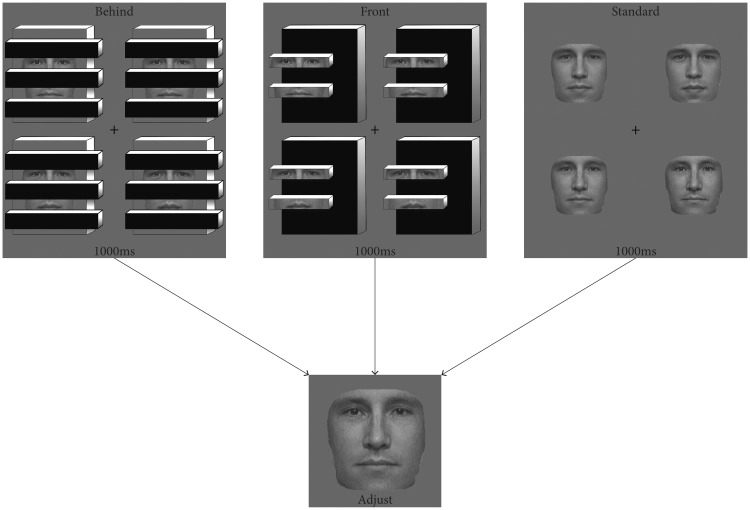
Conditions and procedure for Experiment 1. Observers viewed randomly interleaved conditions and adjusted a test face to match the mean identity of the preceding set. Not shown is a fourth condition, given to 21 participants, composed of inverted faces in the behind condition.

In Experiment 1, we explored the representation of average identity when crowds of faces were visually incomplete. A significant body of research already suggests that we can derive high-level identity information (e.g., Fockert & Wolfenstein, 2009; Haberman et al., 2015; Neumann, Schweinberger, & Burton, 2013), but how much does this depend on having holistic information available to the visual system? We presented observers with sets of faces varying in identity and asked them to report the average identity of a given set. In some of the conditions, faces were partially occluded or partially fragmented (Figure 2). It is already known that face processing (e.g., recognition) is robust to environmental occlusion or noise (e.g., Nakayama, He, & Shimojo, 1995; Sinha, Balas, Ostrovsky, & Russell, 2006), even when key information is obscured from view. Here, we tested whether ensemble processing of partially occluded faces is also robust to visual obstruction. If the ensemble representation of occluded faces does not suffer relative to normal faces, it would suggest ensembles can be represented given incomplete visual information. In addition, it would add to the body of work suggesting that face averaging is a high-level, holistic process and does not depend on piecemeal, featural analysis, or high-fidelity individual item representation (e.g., Haberman & Whitney, 2010; Leib et al., 2014; Rhodes et al., 2018).

Included in this experiment is a critical condition that forces a part-based analysis of sets of faces. Instead of occluders blocking parts of each face, fragments of each face were displayed in the fore—the very same fragments that were part of the background in the occluded condition (Figure 2). In this condition, ensemble face processing should be disrupted, since the benefits of amodal completion are no longer available (Sekuler & Murray, 2001). Taken together, these results would suggest that high-level ensemble processing may operate over inferred visual information.

### Method

#### Participants

Thirty-eight Rhodes College undergraduates, aged 18 to 22 years, participated in this study for either course credit or monetary compensation. The compensation rate was $10 per hour. All participants gave informed consent and had normal or corrected-to-normal vision. This research, and all research described herein, was approved by and conducted in accordance with the institutional review board at Rhodes College.

#### Stimuli and design

Observers were presented with sets of faces varying in identity. Stimuli consisted of 360 linearly interpolated identity morphs, taken from the Harvard Face Database, of three distinct male faces (A-B-C-A), generated using MorphAge software (version 4.1.3, Creaceed). Face morphs were nominally separated from one another in identity units, with each unit corresponding to a degree in morph space. Face identity formed a circular stimulus space spanning 360°. All stimuli in this and future experiments were presented in grayscale using custom scripts developed in Psychophysics toolbox (Brainard, 1997) within MATLAB (Mathworks, Natick, MA).

Three conditions were included: sets of faces behind occluding bars (behind), sets of face fragments presented in the foreground (front), and sets of fully intact faces (standard; see Figure 2). The face pieces presented in the behind and front conditions contained identical face information, only the figure ground relation was flipped. Each face in the standard condition was displayed at 180 × 180 pixels, subtending 5.2° × 5.2° of visual angle. Each face in the behind and front conditions was 259 × 259 pixels, subtending 7.5° × 7.5° of visual angle (note that while the overall size of the image in the standard and front or behind conditions differed, this was done to equate the relative size of the visible facial information on the screen). Sets were followed by a single face in the center of the screen (described later) at 200 × 200, subtending 5.8° × 5.8° of visual angle.

A subset of these observers (*n* = 21) also participated in a fourth condition in which they viewed sets of faces in the behind condition, but inverted (i.e., bars appearing in front of upside down faces). Inverting faces is known to disrupt configural processing (Tanaka & Farah, 1993; Young, Hellawell, & Hay, 1987), and because precise ensemble face perception relies upon such configural information (Haberman & Whitney, 2007, 2009), an inverted ensemble condition serves as a reasonable performance comparison.

Observers were presented with sets of four faces varying in identity (±13 and ± 36 identity units from the mean—this was based on pilot experiments that determined an average just noticeable difference of approximately 26 identity units for these face morphs). The mean of each set was randomly selected on every trial, and then the set of items were centered on that mean.

#### Procedure

On each trial, observers were tasked to report the average identity of a group of faces. Observers, with their heads resting on a chinrest 63 cm from the screen, viewed sets of four faces varying in identity for 1 s. After a 250-ms ISI, a single test face appeared at the center of the screen. The test face was always the original, unobstructed version, regardless of the condition of the preceding set. The starting identity of the test face was chosen at random from the identity wheel. Observers adjusted the test face to match the average identity of the preceding set by moving the mouse along the *x*-axis. As the mouse was moved, the appearance of the face scrolled through the identity wheel. When observers were satisfied with their selection, they pressed the spacebar to lock in their response and begin the next trial.

Prior to the beginning of the experiment, observers performed 24 practice trials in the standard condition, which were discarded from further analysis. The primary task consisted of 80 trials in each of the three conditions, for a total of 240 trials. For those participants who also viewed the inverted faces, there were 60 trials for each condition, for a total of 240 trials.

#### Data availability

All data generated or analyzed for these experiments are available from the corresponding author upon request.

### Results and Discussion

For each observer and condition, the mean absolute error was calculated as an index of average identity precision (i.e., how far away observer responses were from the actual mean of the set). Smaller absolute error indicates greater ensemble precision. Observer’s performance (as indicated by mean absolute error) that was two standard deviations worse than the average overall performance on any condition was excluded from analysis, resulting in the exclusion of two observers, for a total of 36 participants.

**Figure 3.**
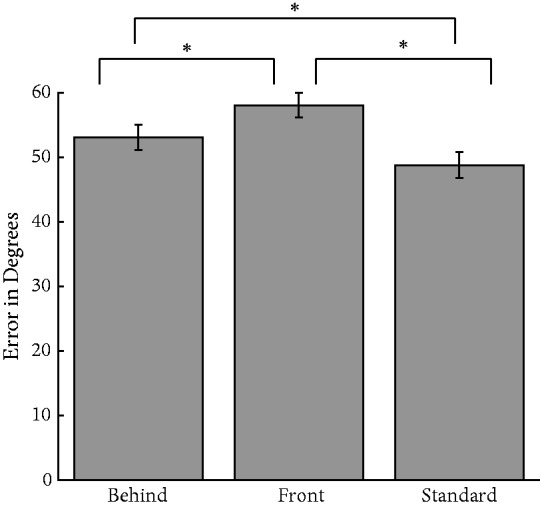
Average absolute error for the three ensemble identity conditions for Experiment 1. Observers were significantly worse in the front condition relative to the standard condition, and performance in the behind condition similarly suffered relative to the standard condition. Error bars indicate standard error of the mean (*SEM*). *p < .05.

A one-way repeated measures analysis of variance (ANOVA) revealed a significant effect of condition, *F*(2, 74) = 18.1, *p* < .0001; η = .33 (Figure 3), where error for the standard condition was smallest (*M*_standard_ = 48.8°), error for the behind condition was in the middle (*M*_behind_ = 53.1°), and error for the front condition was highest (*M*_front_ = 58.0°). Tukey honestly significant difference (HSD) tests revealed that all conditions significantly differed from one another. Although both behind and front conditions differed from the standard condition, the magnitude of the difference for the front condition, where observers did not derive the benefit of amodal completion, was nearly twice that of the behind condition. Thus, while partial removal of facial information did negatively impact ensemble performance, this was exacerbated when the figure ground relation was reversed. This experiment reveals that ensemble perception is robust enough to overcome some, but not all, of the effects of the physical blockage of parts of the face. These deleterious effects are mitigated through configural processing, which is facilitated by amodal completion in the behind condition (Nakayama et al., 1995).

Interestingly, as much as performance suffered in the front condition, it was still significantly better than in the inverted condition—when directly comparing the observers who viewed both conditions—*M*_front_ = 59.9°; *M*_inverted_ = 69.5°; *t*(19) = −3.31, *p* = .004, *η* = .37. Thus, judgments in the front condition are not at floor. Switching the face fragments from background to foreground clearly disrupts configural processing, but not to the same extent that inverting them does.

## Experiment 2

**Figure 4.**
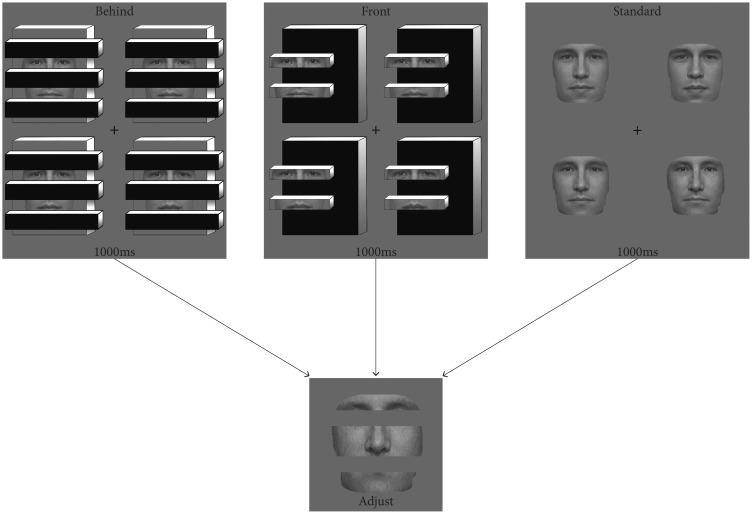
Conditions and procedure for Experiment 2. Observers viewed randomly interleaved conditions and adjusted a fragmented test face containing complementary missing parts to match the mean identity of the preceding set.

Experiment 1 established that ensemble expression information is available even if parts of the faces are blocked from view, although performance suffered to some extent. It is tempting to conclude that people are performing the ensemble calculus over the missing information in their average representation, albeit at reduced precision relative to the standard condition. However, the previous experiment did not explicitly establish *what* information individuals were averaging. In the current experiment, observers viewed the same conditions as described in Experiment 1, but they adjusted a test face that only contained the complementary facial information (Figure 4). Only parts that were missing from the original stimulus were shown during the adjustment phase, thereby testing the quality of the representation of the missing facial information.

### Method

#### Participants

Fifteen Rhodes College undergraduates, aged 18 to 22 years, participated in this study for either course credit or monetary compensation. The compensation rate was $10 per hour. All participants gave informed consent and had normal or corrected-to-normal vision.

#### Stimuli, design, and procedure

Setup for this experiment was nearly identical to that described for Experiment 1. The one major difference was in the test face displayed during the adjustment portion of the trial. Instead of adjusting an intact face to match the perceived mean of the preceding set, observers adjusted a test face containing the complementary missing information (to maintain consistency, this was also true for the standard sets where all information was visible). In our stimuli, forehead, nose, and chin information was absent in the behind and front conditions, and thus that was what was visible to observers during the adjustment phase (Figure 4).

### Results and Discussion

Results were analyzed as described in Experiment 1. Observers whose performance was two standard deviations worse than the average performance for any condition were excluded from analysis, resulting in the exclusion of three observers, for a total of 12 participants.

**Figure 5.**
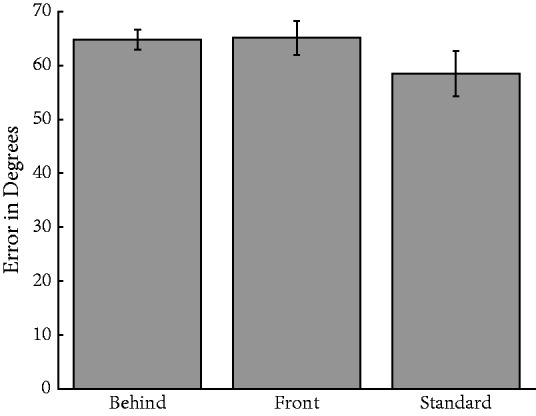
Average absolute error for the three ensemble identity conditions when observers adjusted complementary missing face information for Experiment 2. There was a significant effect of condition at the *p* < .05 level. Error bars indicate standard error of the mean.

Results of this experiment are displayed in Figure 5. There was a significant effect of viewing condition, as revealed by a one-way repeated-measures ANOVA, *F*(2, 22) = 3.91, *p* = .04, η = .26. Performance appears to have suffered to the same extent in both the behind and front conditions relative to the standard conditions (although a post hoc Tukey HSD test revealed no differences among conditions: *M*_behind_ = 64.8; *M*_front_ = 65.1; *M*_standard_ = 58.5). These results suggest that when information is obscured from view, individuals are not explicitly averaging the missing information, or are doing so poorly. Amodal completion may not actually allow the visual system to recreate the missing information, but rather support a *best-guess* heuristic, akin to visual completion. Performance in the standard condition may be marginally better than the front and behind conditions because observers do not have to visually complete any information. Also note that performance in the standard condition suffered in this task relative to Experiment 1, as revealed by a between-subjects *t* test, *t*(48) = − 2.25, *p* = .03, because the relative amount of information available in the test stimulus itself is reduced.

## Experiment 3

This experiment generalizes the findings established in Experiment 1 by exploring high-level, amodal ensemble representations for sets of faces varying in expression. Although substantial evidence points to robust ensemble perceptual abilities for both identity and emotion (Fockert & Wolfenstein, 2009; Haberman & Whitney, 2007, 2010), one cannot assume equivalence given their well-established behavioral and neural independence (Bruce & Young, 1986; Haxby & Gobbini, 2011).

### Method

#### Participants

Twenty Rhodes College undergraduates, aged 18 to 21 years, participated in this study for either course credit or monetary compensation. The compensation rate was $10 per hour. All participants gave informed consent and had normal or corrected-to-normal vision.

#### Stimuli and design

**Figure 6.**
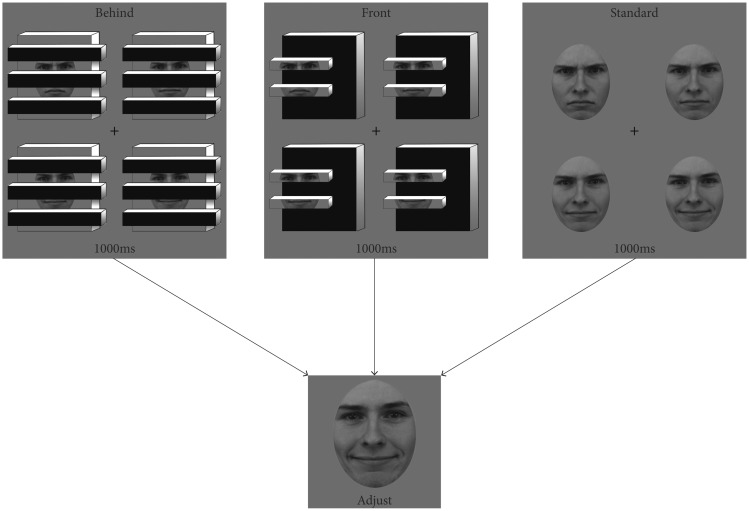
Conditions and procedure for Experiment 3. Observers viewed randomly interleaved conditions and adjusted a test face to match the mean expression of the preceding set.

Observers were presented with sets of faces that differed in emotional expression (Figure 6). In this experiment, the face morphs constituted a circle of 360 expressions (as with the identity stimuli), spanning from angry to happy to sad and back to angry. Faces came from the publicly available Karolinska Directed Emotional Faces database (KDEF; Lundqvist, Flykt, & Öhman, 1998). Distance between two given faces was nominally measured in emotional units. As in Experiment 1, there were three conditions: behind, front, and standard. Faces in the behind and front conditions were 292 × 292 pixels in size, subtending 8.5° × 8.5° degrees of visual angle. Faces in the standard condition were 122 × 158 pixels in size, subtending 3.5° × 4.6° of visual angle. The subsequent test face was 121 × 158, subtending 4.4° × 5.7° of visual angle.

As described earlier, observers were presented with sets of four faces varying in emotional expression (±13 and ±39 emotional units from the mean). The mean of each set was randomly selected on every trial, and then the set items were centered on that mean.

#### Procedure

The task was nearly identical to that described in Experiment 1, but instead of reporting the average identity, observers reported the average expression (Figure 6). As in the previous experiment, observers viewed each set for 1 s, followed by a single test face after a 250-ms ISI. Observers adjusted the test face to match the average expression of the preceding set of faces.

Prior to beginning the experiment, observers performed 24 practice trials in the standard condition, consistent with the number from the previous experiments. For the primary task, observers performed 80 test trials in each of the three conditions, for a total of 240 test trials.

### Results

Performance was assessed as described earlier. Observers whose performance was two standard deviations worse than the average performance for any condition were excluded from analysis, resulting in the exclusion of three observers, for a total of 17 participants.

**Figure 7.**
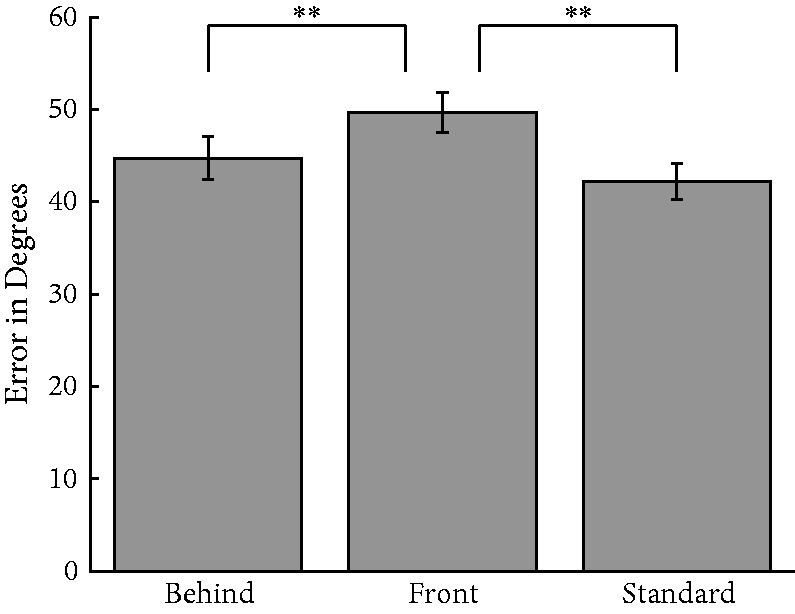
Average absolute error for the three ensemble expression conditions for Experiment 3. Observers were significantly worse in the front condition relative to the standard and behind conditions, while there was no difference in performance between the behind and standard conditions. Error bars indicate standard error of the mean. **p < .01.

Consistent with the previous experiment, a one-way repeated measures ANOVA revealed a significant effect of condition, *F*(2, 32) = 13.7, *p* < .0001; η = .11; Figure 7, where error for the standard condition was smallest (*M*_standard_ = 42.2°), error for the behind condition was in the middle (*M*_behind_ = 44.7°), and error for the front condition was highest (*M*_front_ = 49.7°). A Tukey HSD test revealed the front condition was significantly worse than both the standard and the behind conditions (*p* < .01), consistent with the pattern described in Experiment 1. The post hoc test showed no difference between the standard and behind conditions. However, this does not mean these two conditions are equivalent, only that the benefits of amodal completion extend to processing crowds of emotionally varying faces. It also supports the notion that high-level ensembles may be represented even given an incomplete visual stimulus. Ensemble representation ability is better when the faces amodally complete than when they do not, perhaps facilitated by easier visual completion.

## General Discussion

These experiments reveal that the visual system can extract high-level ensembles even in the presence of incomplete scene information, but at some cost. Observers reported the average expression or average identity for sets of faces that amodally completed behind several occluding bars. The precision of amodally completing sets was only marginally worse for both stimulus domains than when the entirety of the stimulus was presented. This was not a floor effect, as switching the figure ground relationship such that the face fragments were in front of the bars (Figures 2 and 7) further disrupted ensemble performance. Although placing the face fragments in the fore negatively impacted ensemble performance, inverting the amodally completing face stimuli made performance even worse, suggesting that noisy ensemble information was still available from the face fragments.

Finally, Experiment 2 suggested that missing ensemble information obscured by the bars may be partially derived, albeit less precisely. Given the reduction in precision, it reveals that the representation is not based explicitly on the information obscured from view—when observers had to adjust a test face with the complementary missing information to match the mean of the preceding set, performance suffered in both the behind and front conditions. Thus, it does not appear that observers are explicitly representing the missing information but are rather making their inferences as to what was obscured. Given that even basic stimuli, such as familiar shapes or objects, are often variably completed (Boselie, 1988; Van Lier, 1999), it follows that amodally completing faces, where there are even more degrees of freedom of interpretation, are somewhat less precise than fully visible faces (also note that performance was not at floor, as performance on inverted stimuli was still substantially worse).

In the standard condition, observers may have been able to rely upon memory traces of the intact faces to better adjust the fragmented test face. However, adjusting the fragmented test face also negatively impacted the performance in the standard condition relative to when the entire test face was visible. Impairment may also be a function of the relatively less important facial features observers had to visually complete. Most observers, when viewing a face, rely upon specific critical features for identification or emotional recognition (Schyns, Bonnar, & Gosselin, 2002), features that may have been obscured in our task. Thus, deriving a high-fidelity representation of secondary features such as the brow or chin may be of less importance. Future studies should manipulate the kinds of features that amodally complete in the set to test whether amodally completing information critical to identification or emotion recognition may be explicitly represented.

The visual system is often presented with noisy versions of objects in natural scenes, thus in order for object recognition to function it must be robust to common sources of interference. It may not be surprising, then, that one’s ability to recognize a singular face or other object is unimpaired even when it is partially occluded (Nakayama et al., 1995; Sinha et al., 2006). Our results add to this finding, showing only marginal cost to judging sets of partially occluded faces. Overall, this finding suggests that the visual system can infer the ensemble based on exemplar information that is only partially visible.

As noted, viewing fragments in the fore disrupted the ensemble calculus. While observers still had limited access to the ensemble information, the performance decrement suggests a distinct cognitive process from the one operating when faces were amodally completing behind the bars. In other words, the two-dimensional image fragments, while necessary, are insufficient to generate a high-level ensemble representation on their own. This view is consistent with the finding that face and body ensembles are invariant to viewpoint differences (Leib et al., 2014; Sweeney, Haroz, & Whitney, 2012), which would not be possible if only fragmented features were visible. The overall performance benefit in the amodal condition makes sense in light of how often we encounter faces: Behind objects and partially occluded, a situation much more likely to occur than fragmented face pieces.

These experiments dovetail with recent dissertation work examining occluded ensembles for low-level visual stimuli (Lee, unpublished). They represent an early step in understanding what facial components are necessary to generate an accurate ensemble. Ensemble performance suffered a bit in the amodal condition perhaps because too much information was obscured from view— it would be worthwhile to systematically occlude various features of the set to see which elements most affect ensemble representations. Predictions could emerge based on the body of work showing what information is most critical for transmitting a given facial expression (Smith, Cottrell, Gosselin, & Schyns, 2005).

These findings contribute to a growing body of work suggesting ensembles provide a source of stability given the limitations of visual consciousness (Alvarez, 2011; Alvarez & Oliva, 2009; Cohen et al., 2016; Fischer & Whitney, 2011; Haberman & Whitney, 2011). For example, ensemble perception operates rapidly, in as little as 50 ms (Haberman & Whitney, 2009), is generally unaffected by set size (Chong & Treisman, 2003), and occurs beyond the focus of attention (Alvarez & Oliva, 2008), all useful features for a system trying to compensate for limited conscious access. Add to this list, the current set of experiments, which suggest some ensemble information, albeit noisy, is derived even when visual information is altogether missing—arguably a critical component in the effort to create the impression of a complete and holistic visual experience.

## Conclusion

This is one of the first demonstrations that ensemble information may be generated amodally. Furthermore, these amodal representations were revealed in a distinctly high-level example. These results lead one to question the extent to which amodal ensemble representations operate. Future experiments should test other domains within the visual hierarchy, as the system often encounters objects in less-than-ideal viewing conditions. Given the ubiquitous scope and robust nature of ensemble perception, it might be uniquely situated to overcome the challenges of such impoverished (and typical) visual scenes.
